# Three- and six-month efficacy and safety of phentermine in a Mexican obese population 

**DOI:** 10.5414/CP203943

**Published:** 2021-07-08

**Authors:** Maribel Márquez-Cruz, Ashuin Kammar-García, Juan Carlos Huerta-Cruz, Miriam del Carmen Carrasco-Portugal, Lina Marcela Barranco-Garduño, Juan Rodríguez-Silverio, Héctor Isaac Rocha González, Juan Gerardo Reyes-García

**Affiliations:** 1 Sección de Estudios de Posgrado e Investigación, Escuela Superior de Medicina, Instituto Politécnico Nacional. Plan de San Luis y Díaz Mirón s/n, Col. Casco de Santo Tomas, Miguel Hidalgo, Ciudad de México, Mexico, and; 2 Unidad de Investigación en Farmacología, Instituto Nacional de Enfermedades Respiratorias, Ismael Cosio Villegas, Secretaría de Salud, Calzada de Tlalpan 4502, Col. Belisario Domínguez Sección XVI, Tlalpan, 14080, Ciudad de México, México

**Keywords:** long-term phentermine, effectiveness, Mexican, obesity, safety

## Abstract

Objective: Mexico has the second largest prevalence of obesity among adults worldwide, a condition especially affecting the low-income population. There is a pressing need to improve therapeutic options for weight loss. Phentermine is an old and low-cost agent given as an adjuvant therapy for obesity for a 12-week period, at an initial dose of 15 mg or 30 mg. However, there are no precise guidelines on the suitability of both the starting dose and the continuation of treatment for 6 months. The aim of this study was to evaluate the 3- and 6-month efficacy and safety of phentermine in obese Mexican patients to elucidate the aforementioned. Materials and methods: In this prospective, multi-center, open-label study, 932 obese adults received 15 mg or 30 mg phentermine once daily for 6 months. Results: 30 mg phentermine was more effective than 15 mg phentermine in improving anthropometric variables in the 3-month follow-up, but not after completing the 6-month treatment period. Nearly 40% of 3-month non-responders reached a body weight reduction of at least 5% at 6 months. Conversely, ~ 65% and 25% of 3-month responders maintained or improved, respectively, their body weight reduction with long-term phentermine. Potential tolerance as weight regain was ~ 10% from 3 to 6 months. None of the doses increased cardiovascular risk, although mild-to-moderate adverse events were more frequent with 30 mg phentermine. Conclusion: 30 mg phentermine was more effective than 15 mg phentermine after 3 months, but not at 6 months of treatment. An important number of subjects could benefit following the therapy from 3 to 6 months.

**What is known about this subject**

- Phentermine is an effective short-term anti-obesity agent.
- Phentermine dose for adults is 15 mg or 30 mg once daily at physician’s discretion.
- Phentermine should be withdrawn if patient does not respond by 3 months.

**What this study adds**

- 30 mg phentermine was more effective than 15 mg phentermine after 3 months, but not after 6 months of treatment.
- 42% of 3-month non-responders had a reduction in body weight of at least 5% at 6 months.
- 77% of obese patients could benefit from extending phentermine treatment for 3 additional months.

## Introduction 

There is an ongoing pandemic of obesity, a chronic disease associated with a high risk of complications, including diabetes mellitus, heart disease, hypertension, dyslipidemia, osteoarthritis, sleep disorders, and multiple types of cancer. Conversely, a weight loss of at least 5 – 10% can significantly improve health-related outcomes in obese patients. Currently, a 5 – 10% weight loss over a 6-month period is considered a realistic goal with proven health benefit [1]. 

In Mexico, the prevalence of obesity in adults was 31% in 2012 and 33.6% in 2018, when defined as having a body mass index (BMI) of 30 kg/m^2^ or higher. It is projected that by 2050, 54% of men and 37% of women will be obese in Mexico, and obesity-related diseases, such as diabetes, hypertension, stroke, and knee osteoarthritis, among others, will have increased 2-fold [2, 3]. The upward trend in the prevalence of obesity has mainly been attributed to an increased consumption of convenience food and a more sedentary lifestyle. Among obese Mexicans, just 20.2% are diagnosed with the condition, and only 8.0% undergo treatment. In addition, individuals from wealthier households, higher educational settings, and urban areas are more likely to receive treatment for obesity, making evident the need to increase treatment options accessible to groups with lower socio-economic statuses [4]. 

Although lifestyle interventions including diet and physical activity are the cornerstones of anti-obesity therapy, these strategies usually endure poor adherence and a low success rate. Hence, pharmacotherapy is recommended as an adjuvant for patients with a BMI ≥ 30 kg/m^2^ or a BMI ≥ 27 kg/m^2^ if coexistent with obesity-related diseases such as hypertension or type 2 diabetes mellitus [5, 6, 7]. 

Many drugs have been utilized to manage obesity throughout the years, and while some have been withdrawn from the market, others remain despite having been poorly studied [8, 9]. Phentermine, a low-cost agent, is a sympathomimetic amine that acts by inhibiting appetite. It was first approved to aid weight loss in 1959 [10], and it has since become one of the most commonly used weight-loss medications in Mexico and the United States [11, 12]. The current use of 15 mg or 30 mg phentermine for the treatment of obesity is recommended in the short-term (12 weeks), alongside exercise, lifestyle modifications, and caloric restriction [13]. Although the long-term usefulness of phentermine has been suggested in some studies [14, 15], there are no precise guidelines on the suitability of the starting dose, the continuity of treatment for 6 months, nor data on the possibility of tolerance. 

Thus, it is reasonable to make efforts to improve the level of evidence and address unsolved questions of the suitability of old and less expensive drugs. Based on the aforementioned, this study aimed to evaluate the long-term efficacy and safety of phentermine in the treatment of obesity in Mexican patients and to shed light on remaining questions of the optimal use of the drug. 

## Patients and methods 

### Study design 

Efficacy and safety of oral administration of 15 mg or 30 mg of phentermine were assessed in a prospective, multi-center, uncontrolled phase IV, open-label study, which included 932 Mexican participants with ages > 18 years and a BMI ≥ 30 kg/m^2^. Pre-hypertensive and well-controlled hypertensive individuals were allowed to be included in the study if the evaluating physician considered the benefit to be greater than the risk. Exclusion criteria were hypersensitivity to sympathomimetic drugs, use of other weight-loss agents, use of monoamine oxidase inhibitors, psychiatric disorders, renal diseases, closed-angle glaucoma, coronary artery disease, stroke, arrhythmias, congestive heart failure, valvulopathies, history of substance abuse, and pregnant or breast-feeding women. Participants were recruited from 36 clinics located in 12 states of the Mexican Republic between August 2015 and December 2018. This study was approved by the Ethics Committee Unidad Clínica Farmacológica Bioemagno (Code: TER2013) and the Mexican Federal Commission for Protection against Health Risks (Code: CNFV/FI/0147/2013). After obtaining written informed consent from the patients, their baseline characteristics were collected through medical history and clinical examination. Patients were instructed to follow a diet of 1,500 kcal/day and to carry out physical activity for 20 minutes daily. Patients were allocated to receive 15 mg of phentermine or 30 mg of phentermine. Capsules of the drug (terfamex) containing the corresponding dose were prescribed to be taken orally, once every morning before breakfast, for 6 months. All capsules were provided by Productos Medix, S.A. de C.V. (Mexico City, Mexico). 

The primary endpoint was the proportion of patients who experienced at least 5% weight loss with respect to baseline, since this is considered a relevant and achievable goal for patients under treatment with phentermine. Secondary endpoints were weight loss ≥ 10%, changes in BMI, body weight, waist circumference, hip circumference, body fat, visceral fat, systolic blood pressure (SBP), diastolic blood pressure (DBP), and plasma glucose with respect to their baseline values, and safety profile. All measurements were performed at 0, 3, and 6 months of drug treatment. Safety was assessed descriptively by monitoring all adverse events. For anthropometric measurements, standardized scales were used to weigh patients; height was measured with the patient standing upright with the heels together, having back, buttocks, and shoulders in contact with the stadiometer, head erect, facing forward, and with a horizontal gaze. Waist and hip circumference were obtained with a soft measuring tape, placed around the participant at the midpoint just above the superior border of the iliac crest, for waist circumference; and around the widest part in the gluteal region, for hip circumference. Heart rate, SBP, and DBP were measured in the left arm of the patient while seated, using a blood pressure monitor. SBP and DBP were registered as the mean value of 2 consecutive measurements. Body and visceral fat were determined through bioimpedance analysis. In every visit, subjects were interrogated for adverse events, and a physical exam was performed. 

### Data analysis 

Data were analyzed on an intention-to-treat basis. The sample size was calculated to provide a significant difference between two proportions, according to the following equation: 

Equation 1

Where *Z*_α_ at 95% (two-sided) was 1.96; *Z*_β_ at 80%,was 0.84; P_1_ was 0.46 for the number patients who lost 5% of their initial body weight with phentermine 15 mg, according to Aronne et al. [16]; P_2 _was estimated to be 0.66 for phentermine 30 mg; and P, was equal to 0.56. Hence, the calculated sample size was 96 subjects per group. 

Anthropometric, metabolic, and cardiovascular parameters are provided as mean values ± SEM. Demographic data, 5% and 10% body weight loss, and adverse events are given as number of patients and percentages. Adverse events presenting during the follow-up period were categorized in accordance to the Medical Dictionary for Regulatory Activities Terminology (MedDRA) [17]. Tolerance was defined as weight regain to the previous weight percent category. Analysis of variance, followed by Games-Howell’s test for sample sizes of 50 or more individuals, or Tamhane’s test for sample sizes less than 50 individuals, were done to establish the statistical difference of continuous variables. χ^2^ or Fisher’s test was used to analyze categorical variables. The difference was considered statistically significant when p < 0.05. Statistical analysis was carried out using the SPSS 22.0 software. 

## Results 

### Demographic characteristics 

Nearly 80% of patients treated with 15 mg phentermine (400 subjects) or 30 mg phentermine (532 subjects) were women, had class I obesity, were older than 20 years, reported a sedentary lifestyle, were non-smokers, and reported not being daily alcohol drinkers. Comorbidities, including diabetes mellitus, hypertension, thyroid disease, dyslipidemia, metabolic syndrome, and non-alcoholic steatohepatitis, were only present in a small number of patients. All demographic variables were balanced between groups, except for hypertension and diabetes, which were higher in the 15-mg phentermine group (Table 1). Nonetheless, sub-analysis through Fisher’s test revealed that the percentage of hypertensive or diabetic patients who lost at least 5% of their initial body weight was not significantly different between groups at the end of 3-month and 6-month follow up. 

### Efficacy of phentermine 

The effect of phentermine on efficacy parameters is shown in Table 2. Treatment with phentermine led to reductions in weight, which were proportional to the dose and duration of treatment. The group treated with 15 mg phentermine, whose mean baseline weight was 82.7 ± 12.0 kg, showed mean reductions of 4.5 ± 0.2 kg and 6.9 ± 0.4 kg, at 3- and 6-month follow-up, respectively; the group treated with 30 mg phentermine had a mean baseline weight of 86.5 ± 12.0 kg, with reductions of 5.8 ± 0.2 kg and 8.4 ± 0.4 kg, at 3- and 6-month follow-up, respectively. Of note, 30 mg phentermine was statistically superior to 15 mg phentermine in improving most of the anthropometric variables at 3-month follow-up, but not after completing the 6-month treatment (Table 2). The proportion of patients who achieved weight reductions of ≥ 5 – < 10% and ≥ 10% in the 15-mg phentermine group were 49.9 and 16.0% at 3-month follow-up, and 76.7% and 43.7% at 6-month follow-up; whereas the proportion of patients who reached these reductions in the 30-mg phentermine group were 62.3 and 24.1% at 3-month follow-up, and 82.2% and 41.1% at 6-month follow-up, respectively. 15 mg and 30 mg phentermine also improved the rest of the cardiometabolic variables assessed, including body fat, visceral fat, SBP, DBP, and glucose. These parameters were similar in both groups, except for glucose, for which higher reductions were observed with inverse proportionality to the dose and time on drug administration. Phentermine did not increase heart rate or blood pressure with either of the two treatments. 

### Impact from 3- to 6-month administration of 15 mg or 30 mg phentermine 

The impact of oral administration of 3 – 6 months of 15 mg or 30 mg phentermine on the reduction of % weight is shown in Table 3. Participants were grouped into non-responders (< 5%) and responders (≥ 5 – < 10% or ≥ 10%) at 3 months. Daily administration of 15 mg phentermine induced a body weight loss of ≥ 5% – < 10% or ≥ 10% in 46.5% and 2.3% of non-responders, respectively, with a total positive response in the 48.8% of non-responders. Additionally, 15 mg phentermine led to a body weight reduction between ≥ 5 and < 10% in 42.3% and induced a body weight loss ≥ 10% in an additional 44.2% of subjects with a total effectiveness of ≥ 5% in 86.5% of subjects. 100% of subjects with body weight reduction ≥ 10% at 3 months maintained this weight loss from 3 to 6 months. 

Regarding 30 mg of phentermine, it induced a body weight reduction between ≥ 5 and < 10% in 37.7% of non-responders. Moreover, daily administration of 30 mg phentermine plus the advise of exercise and caloric restriction maintained the body weight loss between ≥ 5 and < 10% in 56.6% of responders, and allowed 37.3% of this group to achieve a body weight reduction ≥ 10% with a total effectiveness ≥ 5% in 86.5% of subjects. Subjects with body weight loss ≥ 10% at 3 months maintained this weight loss in 79.4% of cases at 6 months. 

Tolerance (as weight regain) was observed in 13.5% of responders with a body weight loss between ≥ 5 and < 10%, but not with a body weight reduction ≥ 10%, in the 15-mg phentermine group; whereas 6.1% and 20.6% of subjects showed body weight rebound in the ≥ 5% – <10% and ≥ 10% subgroups, respectively, in the 30-mg phentermine arm. 

### Safety of phentermine 

A higher number of adverse events were reported in the 30-mg phentermine group, compared to the 15-mg phentermine group (Table 4). Adverse events occurred in 127 patients in 15-mg phentermine group, and 373 patients in the 30-mg phentermine group at 3-month follow-up. The occurrence of adverse events between the 3-month and 6-month visits were 52 in the 15-mg phentermine group, and 118 in the 30-mg phentermine group. 

The 10 most frequent adverse events were dry mouth, stress, increased appetite, pain in extremity, stretch marks, anxiety, constipation, back pain, polydipsia, and dizziness. Furthermore, dry mouth, stress, increased appetite, anxiety, acanthosis nigricans, polydipsia, and abdominal pain occurred significantly more frequently in patients treated with 30 mg phentermine, compared to 15 mg phentermine. All adverse events were classified as mild (82.1%) or moderate (17.9%), but none as severe. No cardiovascular adverse events were reported. In addition, there were no cases of drug abuse or dependency detected in this study. The drop-out rates were 8.4% (78 subjects) and 66.6% (621 subjects) at 3 and 6 months, respectively. Patient drop-out was for unknown reasons. 

Regarding concomitant drugs at the moment of inclusion in the study, a few patients were taking antihypertensive drugs, including losartan (7), enalapril (5), captopril (4), amlodipine (1), felodipine (1), and chlorthalidone (1); hypoglycemic agents, such as metformin (14), glibenclamide (4), and insulin (2); lipid-lowering drugs, namely pravastatin (2), bezafibrate (6), or atorvastatin (1); or other drugs, such as levothyroxine (4) and multivitamins (4). 

## Discussion 

Obesity is a complex disease that is frequently undertreated in clinical practice. It has been suggested that healthcare practitioners should focus on open discussions and strengthening of realistic weight‐loss goals and outcome assessment according to those goals [18]. Moreover, a realistic goal of 5% or 10% weight reduction could act as a catalyst for further sustained weight loss, in addition to providing clinical benefits such as prevention of diabetes, improved glycemia, and lesser incidence of dyslipidemia, osteoarthritis, gastroesophageal reflux disease, and hypertension as well as reductions in all-cause mortality [1, 19, 20]. Thus, a better understanding of the efficacy, safety, and even the costs of available drugs will allow clinicians to establish rational expectations and goals, when pharmacotherapy is indicated [1]. 

Despite long-time and widespread use of phentermine [21], there are currently no precise guidelines of the suitability of both the starting dose and the continuity of treatment for 6 months, nor data on the rates of tolerance. In this study, we confirmed that phentermine plus the advise of exercise and caloric restriction led to mean reductions in weight that were proportional to the dose administered and duration of treatment, going from 4.5 to 8.4 kg, at 6 months. In addition, we found that the effectiveness rates for 5 and 10% body weight reductions at 3 months were nearly 50% and 15% in the 15-mg phentermine group, which were significantly improved (60% and 25%, respectively) in subjects treated with 30 mg phentermine. Interestingly, continuation of treatment for 6 months similarly increased effectiveness rates to nearly 80% and 40% for 5% and 10% body weight reductions, respectively, with both doses. 

Our findings show the effectiveness rates of phentermine at 3- and 6-month follow-up in the general Mexican population, supporting the expectations of attending physicians who decide to use phentermine. Accordingly, it would be feasible to use 30 mg phentermine when the body weight reduction target is set at 3 months, but the safer 15-mg phentermine dose should be preferred when the body weight reduction target is established at 6 months. In line with our study, other authors have reported similar body weight reductions and efficacy rates for body weight reductions of 5% or more than 10%; although differences in ethnicity, obesity class, duration of treatment, comorbidities, and intermittent or continuous dosing schedules in these studies complicate making direct comparisons [22, 23, 24]. 

Our study also shows that extending treatment from 3 to 6 months is feasible for non-responders or those with body weight reduction goals between ≥ 5 and < 10% at 3 months. In line with this, nearly 50% of non-responders treated with 15 mg phentermine were able to achieve a body weight reduction of at least 5% at 6-month follow-up. In addition, ~ 40% of responders with a body weight loss between ≥ 5 and < 10% at 3 months maintained this weight reduction for 3 months more, and nearly 45% reached a body weight reduction ≥ 10% at 6-month follow-up. Finally, 100% of responders with a body weight loss ≥ 10% maintained this weight loss when treated with 15 mg phentermine for 3 additional months. The corresponding picture in subjects treated with 30 mg phentermine indicated that nearly 40% of non-responders achieved a body weight reduction ≥ 5%, ~ 60% of responders with an initial body weight reduction between ≥ 5% and < 10% maintained this weight reduction, ~ 40% further increased their body weight reduction to ≥ 10%, and almost 80% of responders with a body weight reduction ≥ 10% at 3 months maintained this weight reduction when treated with 30 mg phentermine for 3 additional months. Furthermore, the additional 3-month phentermine treatment continuation could be inconvenient in non-responders with a planned body weight reduction ≥ 10%, whereas nearly 90% of responders could benefit following the treatment from 3 to 6 months, which should be considered according to the initial body weight reduction target. To the best of our knowledge, this is the first study reporting a potential advantage of continuing phentermine treatment from 3 to 6 months; the estimated rates of potential weight regain are ~ 15% with 15 mg phentermine and 25% with 30 mg phentermine, which has traditionally been a worry since phentermine’s mechanism of action is related to norepinephrine release [25]. 

Phentermine plus the advise of exercise and caloric restriction at either the 15-mg or 30-mg dose improved visceral and body fat measurements. Reductions in blood pressure and glycaemia were also observed. Worth noting, heart rate did not increase at 3-month and 6-month follow-up with any of the doses we studied. In this regard, information about the effect that phentermine has on other variables such as blood pressure, heart rate, body fat, or glycaemia is limited; however, in 1 trial using 30 mg phentermine, blood pressure and heart rate were reduced in 3% of participants after 12 weeks of treatment, and these effects were attributed to weight loss rather than other attributable effects of the drug [26]. In addition, 2 observational studies concluded that the continuous use of phentermine for more than 1 year did not increase blood pressure or heart rate in obese patients [14, 15], regardless of being pre-hypertensive or hypertensive [14]. 

The drop-out rate in our study was 8.4% and 66.6% at 3- and 6-month follow-up, respectively, due to unknown reasons. The literature on attrition in the treatment of obesity is heterogeneous, with ranges varying from 10 to 80% depending on the setting and the type of program. Notably, intervention trials reported a mean attrition rate of more than 40% within the first 12 months. These results highlight the importance of a close clinical monitoring in the first weeks of treatment to reduce the attrition rates [27, 28, 29]. In this regard, the literature points out that failure of long-term treatments is frequent in obese patients (mean 30%, with figures reaching up to 80%), frequent causes of attrition being excessive weight-loss expectation, binge eating, and distress [30]. 

In terms of safety, the frequency of adverse events in our study was 3-fold higher in the 30-mg phentermine group compared to the 15-mg phentermine group, which outlines the relationship between higher doses and the frequency of adverse events. The profile of adverse events consisted mainly of gastrointestinal disorders (174), psychiatric disorders (158), and metabolism and nutrition disorders (105). Both phentermine doses were safe and well tolerated since most adverse events were mild, and there were no severe adverse events. This safety profile is in line with those reported in other studies [11, 22, 26]. 

Despite the limitations of our study design, the high drop-out rate, the absence of monthly assessment, and lacking data on the potential phentermine dose titration from 15 to 30 mg phentermine, our results allow us to have a reasonable picture of the general and differential effectiveness rates, safety, and potential weight regain rates of 15 and 30 mg phentermine administered for 3 to 6 months in Mexican subjects. These treatment schemes could be considered by physicians in other countries. 

## Conclusion 

Our data suggest that 30 mg phentermine was more effective than 15 mg phentermine after a 12-week follow-up, but no at 24-week follow-up. In addition, long-term phentermine was safe and well-tolerated at both doses. Approximately 80% subjects could benefit from the continuation of phentermine for 3 additional months. Tolerance to phentermine effect in the 3- to 6-month period was ~10%. 

## Acknowledgment 

This work is part of the PhD dissertation of Maribel Márquez-Cruz. We thank Cecilia Fernandez del Valle-Laisequilla from Laboratorios Medix S.A. de C.V. for her technical assistance. 

## Authors’ contribution 

JGR-G and HIR-G conceptualized the idea and designed the study; MM-C, AK-G, MCC-P, LMB-G, and J-RS performed the acquisition of the data; AK-G, MM-C, JCH-C analyzed the data; and JGR-G, HIR-G, AK-G, and JCH-C interpreted the data for the work. All authors participated in the review, correction, and improvement of the manuscript. All of them approved the final version and agree to be accountable for all aspects of the work in ensuring that questions related to the accuracy or integrity of any part of the work are appropriately investigated and resolved. 

## Funding 

This work was partially supported by Conacyt, PROINNOVA 213481 and 222046. 

## Conflict of interest 

We have no conflict of interest to declare. 

**Table 1.** Baseline demographic characteristics.

Parameter	Phentermine		Total (n = 932)
	15 mg (n = 400)	30 mg (n = 532)	
Sex			
Female	329 (82.3)	411 (77.3)	740 (79.4)
Male	71 (17.8)	121 (22.7)	192 (20.6)
Age, years			
≤ 19	35 (8.8)	32 (6)	67 (7.2)
20 – 29	69 (17.3)	116 (21.8)	185 (19.8)
30 – 39	74 (18.5)	174 (32.7)	248 (26.6)
40 – 49	113 (28.3)	149 (28)	262 (28.1)
50 – 59	84 (21)	49 (9.2)	133 (14.3)
≥ 60	25 (6.3)	12 (2.3)	37 (4)
BMI, kg/m^2^			
30 – 34.9	300 (75)	365 (68.6)	665 (71.4)
35 – 39.9	74 (18.5)	135 (25.4)	209 (22.4)
≥ 40	26 (6.5)	32 (6)	58 (6.2)
Diabetes			
Yes	14 (3.5)	5 (0.9)	19 (2)
No	386 (96.5)	527 (99.1)	913 (98)
Hypertension			
Yes	30 (7.5)	11 (2.1)	41 (4.4)
No	370 (92.5)	521 (97.9)	891 (95.6)
Thyroid disease			
Yes	2 (0.5)	2 (0.4)	4 (0.4)
No	398 (99.5)	530 (99.6)	928 (99.6)
Dyslipidemia			
Yes	13 (3.3)	24 (4.5)	37 (4)
No	387 (96.8)	508 (95.5)	895 (96)
Metabolic syndrome			
Yes	1 (0.3)	1 (0.2)	2 (0.2)
No	399 (99.7)	531 (99.8)	930 (99.8)
Non-alcoholic steatohepatitis			
Yes	12 (3)	8 (1.5)	20 (2.1)
No	388 (97)	524 (98.5)	912 (97.9)
Sedentary lifestyle			
Yes	382 (95.5)	499 (93.8)	881 (94.5)
No	18 (4.5)	33 (6.2)	51 (5.5)
Smokers			
Yes	16 (4)	48 (9)	64 (6.9)
No	384 (96)	484 (91)	868 (93.1)
Daily alcohol consumer			
Yes	0 (0)	2 (0.4)	2 (0.2)
No	400 (100)	530 (99.6)	930 (99.2)

Data expressed as the number of patients (%). BMI = body mass index.

**Table 3.** Impact on effectiveness of 15 mg or 30 mg phentermine from 3 to 6 months.

Previous weight reduction phentermine 15 mg 0 – 3 months	Final weight reduction 3 – 6 months			
	< 5%	≥ 5% – <10%	≥ 10%	Total effectiveness ≥ 5%
< 5% (n = 43)	22 (51.2%)	20 (46.5%)	1 (2.3%)	21 (48.8%)
≥ 5 – <10% (n = 52)	7 (13.5%)	22 (42.3%)	23 (44.2%)	45 (86.5%)
≥ 10% (n = 31)	–	–	31 (100%)	31 (100%)
Previous weight reduction phentermine 30 mg 0 – 3 months	Final weight reduction 3 – 6 months			
	< 5%	≥ 5% – <10%	≥ 10%	Total effectiveness ≥ 5%
< 5% (n = 45)	28 (62.3%)	17 (37.7%)	–	17 (37.7%)
≥ 5 – < 10% (n = 83)	5 (6.1%)	47 (56.6%)	31 (37.3%)	78 (93.9%)
≥ 10% (n = 57)	–	12 (20.6%)	45 (79.4%)	57 (100%)

Data expressed as the number of patients (%).

**Table 2:** Changes in parameters of obese patients who received 15 mg or 30 mg phentermine for 6 months.

Parameter	3 months		6 months	
	Phentermine 15 mg	Phentermine 30 mg	Phentermine 15 mg	Phentermine 30 mg
∆ Weight (kg)	–4.5 ± 0.18*	–5.8 ± 0.18*^,^**	–6.9 ± 0.4*	–8.4 ± 0.4*^,^**
Lost body weight (≥ 5%)^#^	184 (49.9)*	302 (62.3)*^,^**	97 (76.7)*	152 (82.2)*
Lost body weight (≥ 10%)^#^	59 (16.0)*	117 (24.1)*^,^**	55 (43.7)*	76 (41.1)*
∆ BMI (kg/m^2^)	–1.9 ± 0.07*	–2.3 ± 0.07*^,^**	–2.9 ± 0.2*	–3.3 ± 0.2*
∆ Waist circumference (cm)	–5.1 ± 0.3*	–6.2 ± 0.3*^,^**	–8.5 ± 0.6*	–8.2 ± 0.6*
∆ Hip circumference (cm)	–3.6 ± 0.3*	–4.7 ± 0.2*^,^**	–5.8 ± 0.5*	–6.7 ± 0.4*
∆ Body fat (%)	–2.3 ± 0.3*	–2.8 ± 0.3*	–3.3 ± 0.3*	–3.9 ± 0.3*
∆ Visceral fat (%)	–0.6 ± 0.1*	–1 ± 0.07*^,^**	–0.9 ± 0.2*	–1.3 ± 0.1*
∆ Glucose (mg/dL)	–14.4 ± 2.2*	–9.0 ± 2.8*^,^**	–9.7 ± 3.5*	–3.5 ± 2.2*^,^**
∆ SBP (mmHg)	–2.9 ± 0.7*	–2.1 ± 0.6*	–5.4 ± 1.2*	–3.2 ± 1.0*
∆ DPB (mmHg)	–2.0 ± 0.5*	–1.6 ± 0.4*	–3.4 ± 0.9*	–4.0 ± 0.9*
∆ Heart rate (bpm)	–3.20 ± 0.93*	0.43 ± 0.97**	–0.98 ± 1.82	–2.38 ± 1.38

Data expressed as the mean (SEM) or ^#^number of patients (%). *Significantly different with respect to baseline value, and **significantly different with respect to 15 mg phentermine (p < 0.05), as determined by ^#^χ^2^ or analysis of variance, followed by Games-Howell’s test. BMI = body mass index; SBP = systolic blood pressure; DPB = diastolic blood pressure.

**Table 4.** Adverse events reported by patients who received phentermine orally for 6 months.

Adverse event	Phentermine		Total (n = 932)	p-value 15 mg vs. 30 mg
	15 mg (n = 400)	30 mg (n = 532)		
Cardiac disorders				
Dyspnea	1 (0.3)	3 (0.6)	4 (0.4)	0.6
Ear and labyrinth disorders				
Tinnitus	1 (0.3)	4 (0.8)	5 (0.5)	0.3
Gastrointestinal disorders				
Abdominal pain	2 (0.5)	13 (2.4)	15 (1.6)	0.03
Constipation	14 (3.5)	22 (4.1)	36 (3.9)	0.7
Dry mouth	25 (6.3)	74 (13.9)	99 (10.6)	< 0.0001
Dyspepsia	3 (0.8)	8 (1.5)	11 (1.2)	0.4
Nausea	2 (0.5)	6 (1.1)	8 (0.9)	0.5
Vomiting	1 (0.3)	4 (0.8)	5 (0.5)	0.4
General disorders and administration site conditions				
Ataxia	2 (0.3)	0 (0)	2 (0.1)	0.5
Hyperhidrosis	4 (1)	1 (0.2)	5 (0.5)	0.2
Irritability	2 (0.5)	1 (0.2)	3 (0.3)	0.6
Metabolism and nutrition disorders				
Hyperphagia	8 (2)	15 (2.8)	23 (2.5)	0.5
Increased appetite	13 (3.3)	44 (8.3)	57 (6.1)	0.002
Polydipsia	5 (1.3)	20 (3.8)	25 (2.7)	0.02
Musculoskeletal and connective tissue disorders				
Back pain	6 (1.6)	23 (4.4)	29 (3.1)	0.1
Neck pain	1 (0.3)	4 (0.8)	5 (0.6)	0.3
Pain in extremity	14 (3.5)	31 (5.8)	45 (4.8)	0.2
Neoplasms benign, malignant, and unspecified				
Acanthosis nigricans	2 (0.5)	22 (4.1)	24 (2.6)	< 0.0001
Nervous system disorders				
Headache	8 (2.0)	14 (2.6)	22 (2.4)	0.7
Dizziness	2 (0.5)	22 (4.1)	24 (2.6)	< 0.0001
Vertigo	1 (0.3)	0 (0)	1 (0.1)	0.4
Muscle weakness	5 (1.3)	5 (0.9)	10 (1.1)	0.8
Psychiatric disorders				
Anxiety	9 (2.3)	28 (5.3)	37 (4)	0.03
Depression	4 (1)	7 (1.3)	11 (1.2)	0.8
Insomnia	8 (2.0)	15 (2.8)	23 (2.5)	0.5
Nervousness	1 (0.3)	6 (1.1)	7 (0.8)	0.1
Stress	17 (4.3)	63 (11.8)	80 (8.6)	< 0.0001
Respiratory, thoracic, and mediastinal disorders				
Cough	2 (0.5)	4 (0.8)	6 (0.6)	0.8
Hyposmia	1 (0.3)	0 (0)	1 (0.1)	0.4
Sputum increased	0 (0)	2 (0.4)	2 (0.2)	0.5
Renal and urinary disorders				
Polyuria	1 (0.3)	0 (0)	1 (0.1)	0.4
Urinary incontinence	1 (0.3)	0 (0)	1 (0.1)	0.4
Urinary retention	1 (0.3)	1 (0.3)	2 (0.2)	0.9
Bladder spasm	1 (0.3)	2 (0.4)	3 (0.3)	0.7
Skin and subcutaneous tissue disorders				
Stretch marks	11 (2.8)	27 (5.1)	38 (4.1)	0.09
Total	179	491	670	0.0001

Data expressed as the number of patients (%). Adverse events were classified according to the Medical Dictionary for Regulatory Activities Terminology. p-values were obtained with χ^2^.

**Equation 1.**
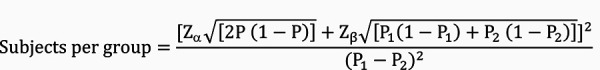
Equation 1

## References

[1] Fruh SM Obesity: Risk factors, complications, and strategies for sustainable long-term weight management. J Am Assoc Nurse Pract. 2017; 29: S3–S14. 29024553 10.1002/2327-6924.12510 PMC6088226

[2] Jensen MD Ryan DH Apovian CM Ard JD Comuzzie AG Donato KA Hu FB Hubbard VS Jakicic JM Kushner RF Loria CM Millen BE Nonas CA Pi-Sunyer FX Stevens J Stevens VJ Wadden TA Wolfe BM Yanovski SZ Jordan HS 2013 AHA/ACC/TOS guideline for the management of overweight and obesity in adults: a report of the American College of Cardiology/American Heart Association Task Force on Practice Guidelines and The Obesity Society. Circulation Circulation. 2014; 129: S102–S138. Erratum in: Circulation. 2014; 129 (Suppl 2): S139-S140. 24222017 10.1161/01.cir.0000437739.71477.ee PMC5819889

[2] Shamah-Levy T Campos-Nonato I Cuevas-Nasu L Hernández-Barrera L Morales-Ruán MDC Rivera-Dommarco J Barquera S [Overweight and obesity in Mexican vulnerable population. Results of Ensanut 100k]. Salud Publica Mex. 2019; 61: 852–865. 31869549 10.21149/10585

[3] Rtveladze K Marsh T Barquera S Sanchez Romero LM Levy D Melendez G Webber L Kilpi F McPherson K Brown M Obesity prevalence in Mexico: impact on health and economic burden. Public Health Nutr. 2014; 17: 233–239. 23369462 10.1017/S1368980013000086 PMC10282205

[4] Pérez-Salgado D Valdés Flores J Janssen I Ortiz-Hernández L Diagnosis and treatment of obesity among Mexican adults. Obes Facts. 2012; 5: 937–946. 23296335 10.1159/000346325

[5] Scheen AJ The future of obesity: new drugs versus lifestyle interventions. Expert Opin Investig Drugs. 2008; 17: 263–267. 10.1517/13543784.17.3.263 18321226

[6] Hainer V Toplak H Mitrakou A Treatment modalities of obesity: what fits whom? Diabetes Care. 2008; 31: S269–S277. 18227496 10.2337/dc08-s265

[7] Delaet D Schauer D Obesity in adults. BMJ Clin Evid. 2011; 2011: 0604. PMC3217730 21411021

[8] Glazer G Long-term pharmacotherapy of obesity 2000: a review of efficacy and safety. Arch Intern Med. 2001; 161: 1814–1824. 11493122 10.1001/archinte.161.15.1814

[9] Powell AG Apovian CM Aronne LJ New drug targets for the treatment of obesity. Clin Pharmacol Ther. 2011; 90: 40–51. 21654742 10.1038/clpt.2011.82

[10] Xia Y Kelton CM Guo JJ Bian B Heaton PC Treatment of obesity: Pharmacotherapy trends in the United States from 1999 to 2010. Obesity (Silver Spring). 2015; 23: 1721–1728. 26193062 10.1002/oby.21136

[11] Bray GA Heisel WE Afshin A Jensen MD Dietz WH Long M Kushner RF Daniels SR Wadden TA Tsai AG Hu FB Jakicic JM Ryan DH Wolfe BM Inge TH The science of obesity management: An Endocrine Society scientific statement. Endocr Rev. 2018; 39: 79–132. 29518206 10.1210/er.2017-00253 PMC5888222

[12] Patel D Pharmacotherapy for the management of obesity. Metabolism. 2015; 64: 1376–1385. 26342499 10.1016/j.metabol.2015.08.001

[13] Colman E Anorectics on trial: a half century of federal regulation of prescription appetite suppressants. Ann Intern Med. 2005; 143: 380–385. 16144896 10.7326/0003-4819-143-5-200509060-00013

[14] Hendricks EJ Greenway FL Westman EC Gupta AK Blood pressure and heart rate effects, weight loss and maintenance during long-term phentermine pharmacotherapy for obesity. Obesity (Silver Spring). 2011; 19: 2351–2360. 21527891 10.1038/oby.2011.94

[15] Lewis KH Fischer H Ard J Barton L Bessesen DH Daley MF Desai J Fitzpatrick SL Horberg M Koebnick C Oshiro C Yamamoto A Young DR Arterburn DE Safety and Effectiveness of Longer-Term Phentermine Use: Clinical Outcomes from an Electronic Health Record Cohort. Obesity (Silver Spring). 2019; 27: 591–602. 30900410 10.1002/oby.22430

[16] Aronne LJ Wadden TA Peterson C Winslow D Odeh S Gadde KM Evaluation of phentermine and topiramate versus phentermine/topiramate extended-release in obese adults. Obesity (Silver Spring). 2013; 21: 2163–2171. 24136928 10.1002/oby.20584

[17] The National Center for Biomedical Ontology. Medical Dictionary for Regulatory Activities Terminology (MedDRA). November 2019; http://bioportal.bioontology.org/ontologies/MEDDRA?p=summary.

[18] Bray G Look M Ryan D Treatment of the obese patient in primary care: targeting and meeting goals and expectations. Postgrad Med. 2013; 125: 67–77. 24113665 10.3810/pgm.2013.09.2692

[19] Velazquez A Apovian CM Updates on obesity pharmacotherapy. Ann N Y Acad Sci. 2018; 1411: 106–119. 29377198 10.1111/nyas.13542

[20] Kritchevsky SB Beavers KM Miller ME Shea MK Houston DK Kitzman DW Nicklas BJ Intentional weight loss and all-cause mortality: a meta-analysis of randomized clinical trials. PLoS One. 2015; 10:e0121993. 25794148 10.1371/journal.pone.0121993 PMC4368053

[21] Hendricks EJ Rothman RB Greenway FL How physician obesity specialists use drugs to treat obesity. Obesity (Silver Spring). 2009; 17: 1730–1735. 19300434 10.1038/oby.2009.69

[22] Haddock CK Poston WS Dill PL Foreyt JP Ericsson M Pharmacotherapy for obesity: a quantitative analysis of four decades of published randomized clinical trials. Int J Obes Relat Metab Disord. 2002; 26: 262–273. 11850760 10.1038/sj.ijo.0801889

[23] Kang JG Park CY Kang JH Park YW Park SW Randomized controlled trial to investigate the effects of a newly developed formulation of phentermine diffuse-controlled release for obesity. Diabetes Obes Metab. 2010; 12: 876–882. 20920040 10.1111/j.1463-1326.2010.01242.x

[24] Li Z Hong K Yip I Huerta S Bowerman S Walker J Wang H Elashoff R Go VL Heber D Body weight loss with phentermine alone versus phentermine and fenfluramine with very-low-calorie diet in an outpatient obesity management program: a retrospective study. Curr Ther Res Clin Exp. 2003; 64: 447–460. 24944395 10.1016/S0011-393X(03)00126-7 PMC4053043

[25] Li Z Maglione M Tu W Mojica W Arterburn D Shugarman LR Hilton L Suttorp M Solomon V Shekelle PG Morton SC Meta-analysis: pharmacologic treatment of obesity. Ann Intern Med. 2005; 142: 532–546. 15809465 10.7326/0003-4819-142-7-200504050-00012

[26] Vallé-Jones JC Brodie NH O’Hara H O’Hara J McGhie RL A comparative study of phentermine and diethylpropion in the treatment of obese patients in general practice. Pharmatherapeutica. 1983; 3: 300–304. 6844367

[27] Moroshko I Brennan L O’Brien P Predictors of dropout in weight loss interventions: a systematic review of the literature. Obes Rev. 2011; 12: 912–934. 21815990 10.1111/j.1467-789X.2011.00915.x

[28] Dansinger ML Gleason JA Griffith JL Selker HP Schaefer EJ Comparison of the Atkins, Ornish, Weight Watchers, and Zone diets for weight loss and heart disease risk reduction: a randomized trial. JAMA. 2005; 293: 43–53. 15632335 10.1001/jama.293.1.43

[29] Colombo O Ferretti VV Ferraris C Trentani C Vinai P Villani S Tagliabue A Is drop-out from obesity treatment a predictable and preventable event? Nutr J. 2014; 13: 13. 24490952 10.1186/1475-2891-13-13 PMC3914843

[30] Michelini I Falchi AG Muggia C Grecchi I Montagna E De Silvestri A Tinelli C Early dropout predictive factors in obesity treatment. Nutr Res Pract. 2014; 8: 94–102. 24611111 10.4162/nrp.2014.8.1.94 PMC3944162

